# Incidence and risk factors for recurrence after surgical treatment of rhegmatogenous retinal detachment: a retrospective cohort study

**DOI:** 10.1186/s40942-025-00680-7

**Published:** 2025-05-22

**Authors:** Abdelrahman M. Tawfik, Ahmed Mohamed Eweidah, Rawan Adel Hassanien, Shrouk F. Mohamed, Rawan Ashraf Kasem, Mohammed Ghoneem

**Affiliations:** 1 Faculty of Medicine, Alexandria University, Alexandria, Egypt; 2 Ophthalmology Department, Faculty of Medicine, Alexandria University, Alexandria, Egypt

**Keywords:** Rhegmatogenous retinal detachment, Recurrence, Pars plana vitrectomy, Scleral buckling, Risk factors, Proliferative vitreoretinopathy

## Abstract

**Background:**

Rhegmatogenous retinal detachment (RRD) is a vision-threatening ophthalmic emergency requiring prompt surgical intervention. Despite advancements in surgical techniques, recurrence remains a significant challenge, leading to additional surgeries and poorer visual outcomes. This study aimed to evaluate the incidence and risk factors for RRD recurrence following surgical repair in an Egyptian tertiary care setting.

**Methods:**

A retrospective cohort study was conducted at Alexandria Main University Hospital, Egypt, including 134 patients who underwent RRD surgery (pars plana vitrectomy [PPV] or scleral buckling [SB]) between March and September 2023. Demographic, clinical, and surgical variables were evaluated. Recurrence was defined as anatomical detachment after initial surgical success within a 6-month follow-up period. Statistical analyses included chi-square tests and multivariate logistic regression to identify independent risk factors.

**Results:**

The recurrence rate was 24.6%, with early recurrence (≤ 6 weeks) occurring in 14.9% of cases. PPV had a significantly higher recurrence rate (34.8%) compared to SB (19.3%) (*p* = 0.049). Univariate analysis identified right eye laterality (*p* = 0.02), high myopia (*p* = 0.015), proliferative vitreoretinopathy (PVR) (*p* < 0.001), and ocular comorbidities (*p* = 0.018) as significant risk factors. Multivariate analysis confirmed right eye laterality (OR: 3.7, *p* = 0.016), high myopia (OR: 0.34, *p* = 0.04), and PVR (OR: 0.15, *p* = 0.005) as independent predictors. Surgeon experience significantly influenced outcomes in univariate analysis (*p* = 0.001), but not in adjusted models.

**Conclusions:**

RRD recurrence remains prevalent occurring in nearly one-quarter of repaired RRD cases, predominantly within the early postoperative period. Surgical technique, laterality, and ocular characteristics significantly impacted recurrence risk. These findings highlight the need for individualized surgical planning and enhanced surveillance in high-risk patients, particularly during the critical first postoperative weeks.

## Background

Rhegmatogenous retinal detachment is a vision-threatening ophthalmic emergency characterized by the separation of the neurosensory retina from the underlying retinal pigment epithelium, typically caused by a retinal tear or break that allows subretinal fluid to accumulate [1]. It is one of the most common forms of retinal detachment and represents one of the significant causes of visual morbidity worldwide [2]. If left untreated, rhegmatogenous retinal detachment (RRD) may lead to irreversible loss of vision. Thus, early diagnosis and intervention remain imperative in preserving useful vision [3, 4].

Over the past 80 years, RRD treatment has evolved significantly, focusing on surgical techniques to close retinal breaks and reattach the retina [5]. Scleral buckling (SB), introduced in the 1950s, achieved success rates of nearly 90% and was the primary treatment for decades. In the 1970s, pars plana vitrectomy (PPV) emerged, alongside intraocular gases like sulphur hexafluoride (SF6) to temporarily seal breaks until permanent adhesions form. It turned out that in complex cases, such as proliferative vitreoretinopathy or trauma, silicone oil (SO) worked as an alternative. More recently, micro-incisional techniques and improved instrumentation have favored PPV as the first-line surgical approach in the management of primary and complex RRD, thus minimizing complications and improving visual outcomes [2, 6]. These have ensured the full potential of modern techniques of surgery and have helped retinal surgeons of rhegmatogenous retinal detachment with a very high success rate [7].

Recurrence of RRD poses a heavy physical, psychological, and financial burden on patients. It requires repeat surgeries and prolongs recovery. Poorer visual outcomes have also been linked with recurrence, attributed to complicating factors such as PVR and macular involvement [8]. Various studies show overall recurrence rates between 5% and 37.3%, while SO tamponade presents recurrence rates from 21.4% up to77.0%. Additionally, 34% of patients had a recurrence following SO removal [9]. Inferior RRDs are particularly problematic; the buoyancy of SO is often unable to block inferior breaks, thus permitting fluid accumulation and progression of PVR [9, 10]. Occasionally, SB has been performed to address the problem of recurrence; however, efficacy remains a matter of debate. Early recurrence occurs within 6 weeks after surgery, whereas late recurrence rates were reported to be as low as 0–4.0% at 4–6 months after the primary SB or PPV [9, 11]. Although recent advances have led to primary reattachment rates of over 90% in uncomplicated cases, recurrent RRD, often secondary to PVR, remains among the most common causes of repeated surgeries [12, 13].

Various studies have identified risk factors associated with RRD recurrence with conflicting reports. Variably reported risk factors include high myopia, multiple or large retinal breaks, incomplete closure of the break intraoperatively, and PVR as being associated with a higher incidence of recurrence. Patient-related variables may include age and sex, and surgical variables and surgeon experience may also influence outcomes [4, 8, 11, 14–16]. Nevertheless, these factors’ relative contributions and interactions have not yet been fully identified; thus, calling for the implementation of additional studies in the present area.

The primary aim of this study is to evaluate the incidence of RRD recurrence following surgical treatment, while the secondary aim was to identify demographic and clinical risk factors associated with recurrence.

## Methods

### Study design and setting

This retrospective observational cohort study was approved by the Institutional Review Board of the Faculty of Medicine, Alexandria University, and adhered to the principles of the Declaration of Helsinki Patient confidentiality was maintained by de-identifying all data. Informed consent was waived due to the retrospective nature of the study.

The study was conducted at conducted at Alexandria Main University Hospital, a tertiary care center in Alexandria, Egypt. Patients who underwent surgical repair of primary RRD between March 1, 2023, and September 30, 2023, were included. Cases were screened based on surgical procedures and clinical documentation.

### Inclusion and exclusion criteria

The primary inclusion criteria were patients who achieved initial anatomical retinal reattachment following primary RRD repair using either PPV or SB during the specified period. Also, a minimum postoperative follow-up duration of six months was required.

Exclusion criteria included patients with tractional or exudative retinal detachments, primary surgical failures (persistent detachment following initial surgery), patients undergoing other ocular surgeries at the same time of RRD repair, pediatric patients under the age of 18 years, surgeries performed outside the study center, and incomplete clinical records. Patients with traumatic etiologies, or combined tractional detachments were excluded.

### Patient selection and characteristics of surgical technique

Preoperative assessment was done for the patients. Patients were allocated to PPV or SB based on standardized clinical indications, aiming to align with current best practice guidelines. PPV was preferred in pseudophakic eyes, cases with multiple or posterior breaks, significant media opacity, or the presence of vitreous hemorrhage. SB was selected for phakic patients presenting with localized anterior retinal breaks and minimal vitreoretinal traction.

Pneumatic retinopexy was not considered in this study, including for superior breaks, in accordance with institutional protocol prioritizing SB or PPV to maximize single-procedure success rates. None of the cases in our sample required supplementary scleral buckling during PPV. The surgical approach was determined based on preoperative evaluation and confirmed intraoperatively by experienced vitreoretinal surgeons.

For PPV, a standard 23-gauge three-port vitrectomy was performed, including core vitrectomy, induction of posterior vitreous detachment, when necessary, meticulous vitreous base shaving, endodiathermy marking of retinal breaks, drainage of subretinal fluid using perfluorocarbon liquid (PFCL), and retinopexy with endolaser photocoagulation or cryotherapy. Membrane peeling, retinotomy, or retinectomy was performed as required to manage vitreoretinal traction. Internal tamponade was achieved using either silicone oil (2000 cSt) or expansile intraocular gas, depending on intraoperative assessment.

In cases of SB, retinal breaks were localized using indirect ophthalmoscopy, and a silicone exoplant with an encircling band was placed for better scleral indentation, secured with sutures; retinopexy was achieved via transscleral laser or cryotherapy. Adjunctive intraoperative maneuvers, including internal limiting membrane (ILM) peeling, additional PFCL use, or subretinal membrane removal, were selectively applied based on intraoperative findings.

### Data collection methods

A retrospective review of medical records was conducted. Data was collected from records maintained by doctors and clinical audit teams and put into a standardized spreadsheet.

The following data were collected:

- Demographic characteristics: age and sex.
- Surgical details: date of surgery, surgeon experience level, laterality (right or left eye), surgical technique employed (PPV or SB).
- Clinical features: smoking history, family history of retinal detachment, presence of high myopia (≥ -6.00 diopters), lens status (phakic, pseudophakic, aphakic), presence of PVD, PVR status, number and location of retinal breaks, macular involvement, and extent of detachment (by number of quadrants or total detachment).
- Recurrence assessment: recurrent retinal detachment was classified as early recurrence (within six weeks) or late recurrence (beyond six weeks postoperatively), based on clinical examination and imaging (optical coherence tomography or fundus photography).

### Outcome measures

The primary outcome was the incidence of recurrent retinal detachment following surgical repair. Recurrence was defined as the reappearance of retinal detachment in the same eye after initial anatomical success, confirmed by clinical examination and imaging (e.g., optical coherence tomography or fundus photography). Secondary outcomes included identification of risk factors associated with recurrence.

### Statistical analysis

Jamovi statistical analysis software (v.2.3) was used to perform statistical analysis. Descriptive statistics summarized demographic and clinical characteristics. Continuous variables were reported as mean ± standard deviation (SD) or median (interquartile range). Categorical variables were expressed as frequencies and percentages. The rate of recurrent retinal detachment was calculated as the proportion of patients experiencing recurrence over the follow-up period.

Univariate analysis was conducted using chi-square tests to identify potential risk factors associated with recurrence.

A multivariable logistic regression model was used to determine independent risk factors for recurrent retinal detachment, helping control for confounding factors. Factors known to influence retinal detachment outcomes (e.g., lens status, myopia, PVR grade, macular involvement) were included in the adjustment models. Results were presented as odds ratios (OR) with 95% confidence intervals (CI), and a *p*-value < 0.05 was considered statistically significant.

## Results

### Study population and baseline characteristics

**Fig. 1:**
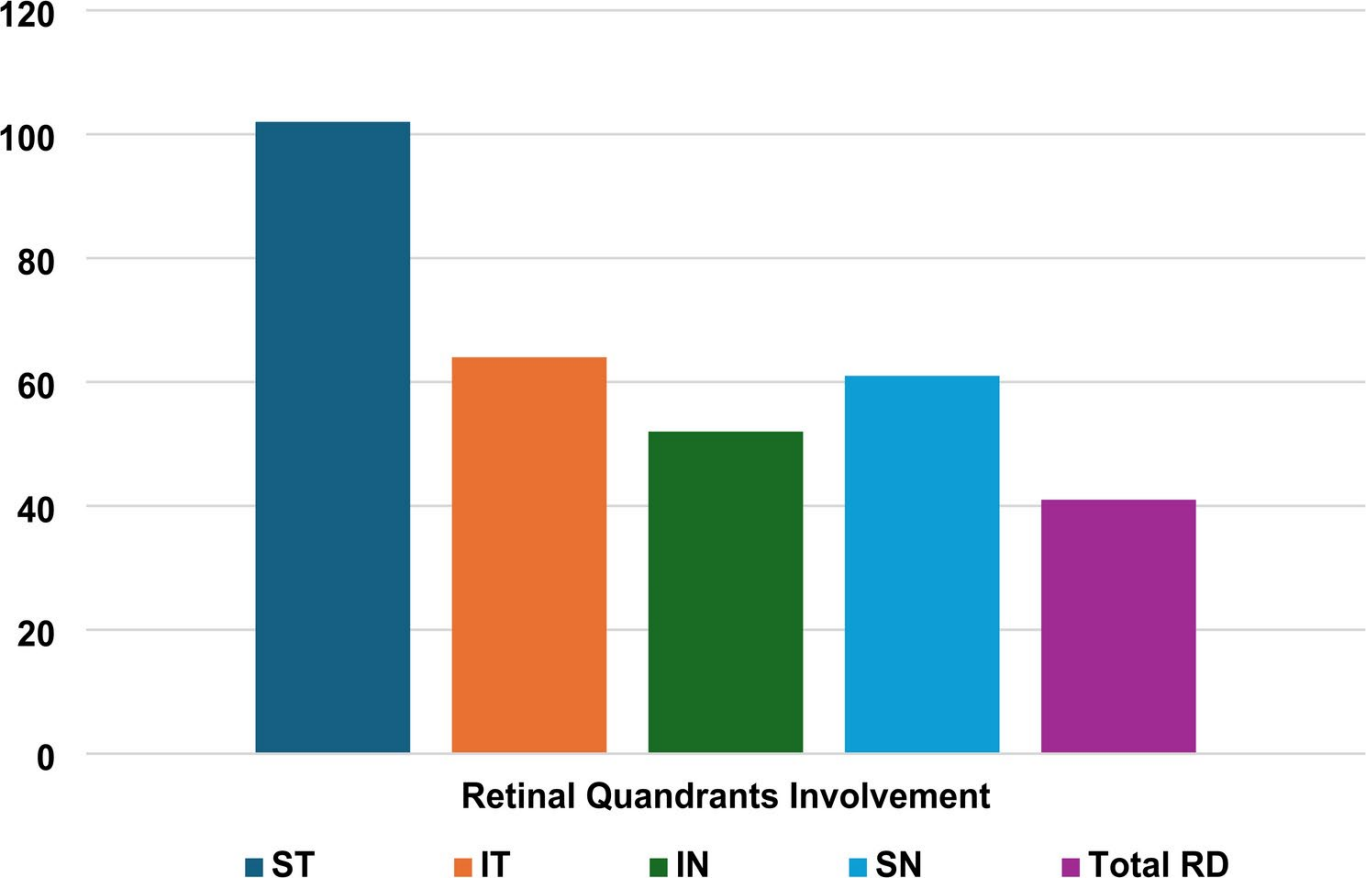
Bar chart showing number of patients where each retina quadrant is involved

This study analyzed data from 134 patients with a mean age of 48.87 ± 13.13. The sample included 91 (67.9%) males and 43 (32.1%) females. Surgical procedures were performed by eight different surgeons with variable levels of experience and numbers of surgeries, ranging from senior residents to professors at our tertiary university hospital. In our cohort, pars plana vitrectomy was performed on 46 (34.3%) of the cases, while the scleral buckle technique was performed in 88 (65.7%). The diseased eye was the right eye in 74 (55.2%) cases and the left eye in 60 (44.8%). The family history of retinal detachment was reported by 11 (8.2%) cases, and 83 (61.9%) of the sample reported active smoking. High myopia of -6 diopters or less was observed in 53 (39.6%) of the cases. Regarding lens status, 94 (70.1%) of the lenses were phakic, 36 (26.9%) were pseudophakic, and 4 (3.0%) were aphakic. Other ocular comorbidities were reported by 29 (21.6%) of patients, including glaucoma, epiretinal membrane, amblyopia, ocular hypertension, uveitis, macular degeneration, and/or retinopathies. Total retinal detachment was observed in 41 (30.6%) patients, while the most affected quadrant was the superior-temporal quadrant with 102 (76.1%) cases (Figure 1). (Table 1) shows the demographics and characteristics of the eyes included in the study.

**Table 1:** Baseline characteristics of the sample

Variable		Total (*N* = 134)
Age	Mean (SD)	48.9 (13.1)
	Range	18.0–83.0
Sex	Male	91 (67.9%)
	Female	43 (32.1%)
Recurrence of RD	Yes	33 (24.6%)
Early Recurrence (6 weeks)	Yes	20 (14.9%)
Late Recurrence (6 months)	Yes	13 (9.7%)
Surgeon Grade	Resident	56 (41.8%)
	Assistant Lecturer	71 (53.0%)
	Lecturer	5 (3.7%)
	Professor	2 (1.5%)
Laterality	Right	74 (55.2%)
	Left	60 (44.8%)
Surgical technique	Pars Plana Vitrectomy	46 (34.3%)
	Scleral Buckle	88 (65.7%)
Family History	Yes	11 (8.2%)
Smoking	Smoker	83 (61.9%)
	Non-smoker	51 (38.1%)
High myopia	> - -6.0 diopters	81 (60.4%)
	≤ − 6.0 diopters	53 (39.6%)
PVD	Yes	55 (41.0%)
	Can not be assessed	8 (6.0%)
PVR	Yes	20 (14.9%)
Lens Status	Phakic	94 (70.1%)
	Pseudophakic	36 (26.9%)
	Aphakic	4 (3.0%)
No. of retinal breaks	1	94 (70.1%)
	2	24 (17.9%)
	3	15 (11.2%)
	6	1 (0.7%)
Macular status	Off	121 (90.3%)
	On	13 (9.7%)
Ocular comorbidities	Yes	29 (21.6%)
Quadrants involvement	Superior-temporal	102 (76.1%)
	Inferior-temporal	64 (47.8%)
	Inferior-nasal	52 (38.8%)
	Superior-nasal	61 (45.5%)
Total detachment	Yes	41 (30.6%)

### Incidence of recurrence

**Fig. 2:**
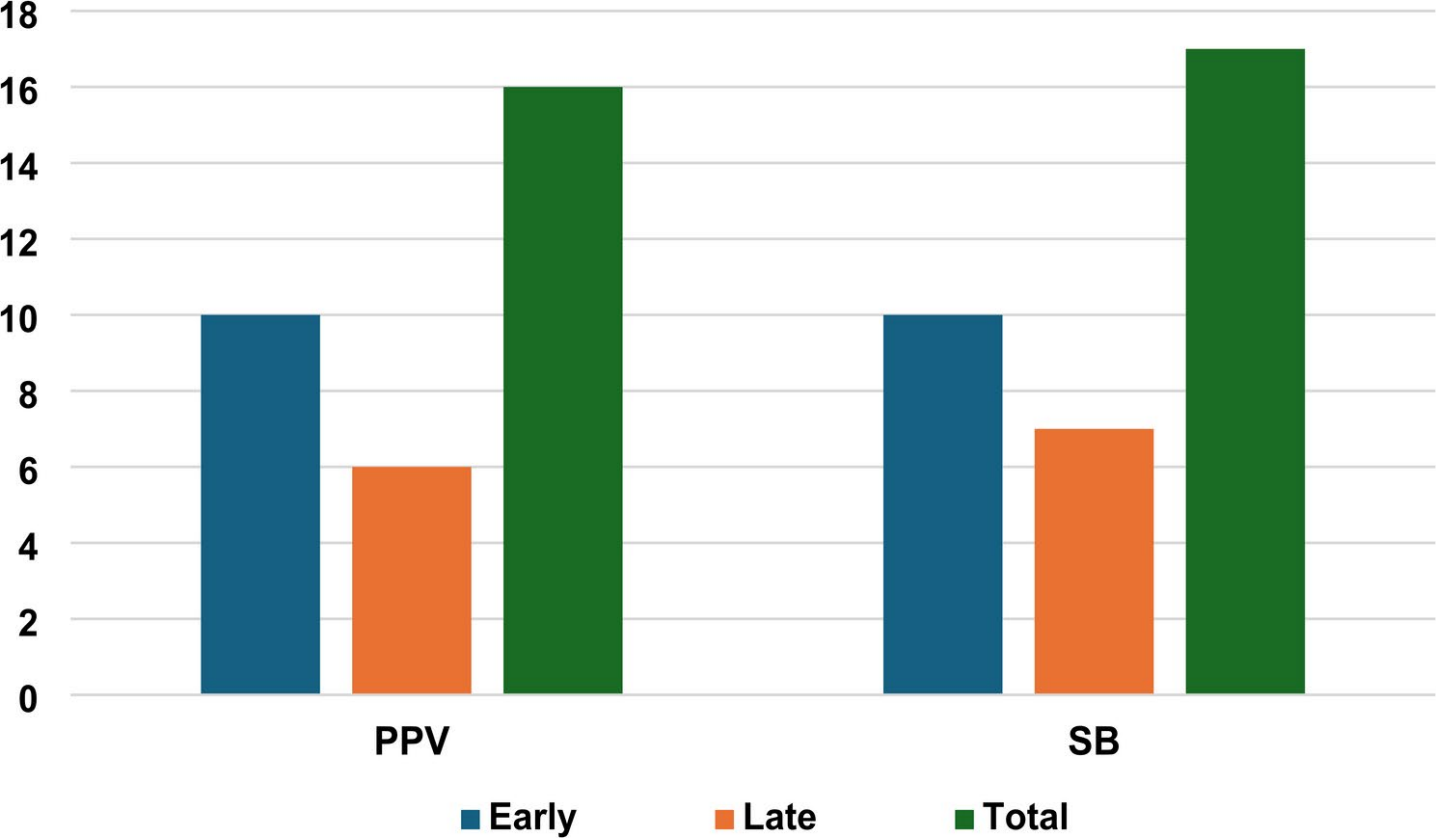
Comparison of early and late recurrence rates using PPV and SB

Eyes were followed up for a duration of 6 months post-surgery. The total rate of recurrence observed in our cohort was 33/134 (24.6%). A total of 20 cases (14.9%) experienced early recurrence in the first 6 weeks after performing surgery, while 13 cases (9.7%) developed late recurrence during a period of 6 months following surgery. Eyes undergoing pars plana vitrectomy experienced a significantly higher rate of recurrence, 16 (34.8%), compared to those treated by the scleral buckle technique, 17 (19.3%) (*p* = 0.049) (Figure 2) (Table 2).

**Table 2:** Recurrence rates for each surgical technique

	PPV (*N* = 46)	SB (*N* = 88)	Total (*N* = 134)	*p*-value
**Total Recurrence**				**0.049** ^**1**^
No	30 (65.2%)	71 (80.7%)	101 (75.4%)	
Yes	16 (34.8%)	17 (19.3%)	33 (24.6%)	
**Early Recurrence (6 weeks)**				0.110^1^
No	36 (78.3%)	78 (88.6%)	114 (85.1%)	
Yes	10 (21.7%)	10 (11.4%)	20 (14.9%)	
**Late Recurrence (6 months)**				0.345^1^
No	40 (87.0%)	81 (92.0%)	121 (90.3%)	
Yes	6 (13.0%)	7 (8.0%)	13 (9.7%)	

1. Pearson’s Chi-squared test

### Risk factor analysis

In univariate analysis, statistical significance in relation to primary RD recurrence were found for the following variables: the primary operating surgeon (*p* = 0.001), right eyes compared to left eyes (*p* = 0.02), high myopia (*p* = 0.015), the presence of proliferative vitreoretinopathy (*p* < 0.001), and the presence of other ocular comorbidities (*p* = 0.018).

Particularly, the rate of recurrence of RD in right eyes was 32.4% compared to 15% in left eyes. Highly myopic eyes demonstrated a higher rate of recurrence (35.8%) compared to low myopic eyes (17.3%). Additionally, 55% of recurrent cases suffered from PVR compared to only 19.2% of successful cases. The recurrence rate in eyes with other ocular comorbidities was 41.4% compared to 20% of those without. Moreover, surgeon variability impacted recurrence rates, where surgeon 1 showed the lowest recurrence rate (6%) while surgeon 3 demonstrated the highest rate (58%).

On the other hand, exploring variables potentially associated with redetachment, no significant relation was found for the following factors: age, sex, family history, smoking, posterior vitreous detachment, lens status, number of retinal breaks, and macular status. Males showed a higher recurrence rate compared to females (27.5% vs. 20.9%) (*p* = 0.495). Smokers had a higher recurrence rate compared to non-smokers (29.5% vs. 22.9%) (*p* = 0.552). Similarly, patients with PVD also showed a higher recurrence rate (29% vs. 22.5%) (*p* = 0.589). (Table 3)

**Table 3:** Univariate analysis of recurrence with recorded risk variables

	Attachment (*N* = 101)	Recurrent Detachment (*N* = 33)	Total (*N* = 134)	*p*-value
**Age**				0.633^1^
Mean (SD)	49.4 (12.8)	47.3 (14.1)	48.9 (13.1)	
Range	18.0–83.0	15.0–67.0	15.0–83.0	
**Sex**				0.495^2^
Male	67.0 (66.3%)	24.0 (72.7%)	91.0 (67.9%)	
Female	34.0 (33.7%)	9.0 (27.3%)	43.0 (32.1%)	
**Surgeon**				**0.001** ^**2**^
1	32.0 (31.7%)	2.0 (6.1%)	34.0 (25.4%)	
2	27.0 (26.7%)	12.0 (36.4%)	39.0 (29.1%)	
3	7.0 (6.9%)	10.0 (30.3%)	17.0 (12.7%)	
4	16.0 (15.8%)	2.0 (6.1%)	18.0 (13.4%)	
5	9.0 (8.9%)	1.0 (3.0%)	10.0 (7.5%)	
6	4.0 (4.0%)	1.0 (3.0%)	5.0 (3.7%)	
7	4.0 (4.0%)	4.0 (12.1%)	8.0 (6.0%)	
8	2.0 (2.0%)	1.0 (3.0%)	3.0 (2.2%)	
**Surgeon Grade**				0.841^2^
Resident	43.0 (42.6%)	13.0 (39.4%)	56.0 (41.8%)	
Assistant Lecturer	53.0 (52.5%)	18.0 (54.5%)	71.0 (53.0%)	
Lecturer	1.0 (1.0%)	1.0 (3.0%)	2.0 (1.5%)	
Professor	4.0 (4.0%)	1.0 (3.0%)	5.0 (3.7%)	
**Laterality**				**0.020** ^**2**^
Right	50.0 (49.5%)	24.0 (72.7%)	74.0 (55.2%)	
Left	51.0 (50.5%)	9.0 (27.3%)	60.0 (44.8%)	
**Surgical technique**				**0.049** ^**2**^
Pars Plana Vitrectomy	30.0 (29.7%)	16.0 (48.5%)	46.0 (34.3%)	
Scleral Buckle	71.0 (70.3%)	17.0 (51.5%)	88.0 (65.7%)	
**Family History**				0.832^2^
No	93.0 (92.1%)	30.0 (90.9%)	123.0 (91.8%)	
Yes	8.0 (7.9%)	3.0 (9.1%)	11.0 (8.2%)	
**Smoking**				0.552^2^
Smoker	64.0 (63.4%)	19.0 (57.6%)	83.0 (61.9%)	
Non-smoker	37.0 (36.6%)	14.0 (42.4%)	51.0 (38.1%)	
**High myopia**				**0.015** ^**2**^
> − 6.0 diopters	67.0 (66.3%)	14.0 (42.4%)	81.0 (60.4%)	
≤ − 6.0 diopters	34.0 (33.7%)	19.0 (57.6%)	53.0 (39.6%)	
**PVD**				0.589^2^
No	56.0 (55.4%)	15.0 (45.5%)	71.0 (53.0%)	
Yes	39.0 (38.6%)	16.0 (48.5%)	55.0 (41.0%)	
Connot be assessed	6.0 (5.9%)	2.0 (6.1%)	8.0 (6.0%)	
**PVR**				**< 0.001** ^**2**^
No	92.0 (91.1%)	22.0 (66.7%)	114.0 (85.1%)	
Yes	9.0 (8.9%)	11.0 (33.3%)	20.0 (14.9%)	
**Lens Status**				0.146^2^
Phakic	75.0 (74.3%)	19.0 (57.6%)	94.0 (70.1%)	
Pseudophakic	24.0 (23.8%)	12.0 (36.4%)	36.0 (26.9%)	
Aphakic	2.0 (2.0%)	2.0 (6.1%)	4.0 (3.0%)	
**No. of retinal breaks**				0.301^2^
1	73.0 (72.3%)	21.0 (63.6%)	94.0 (70.1%)	
3	11.0 (10.9%)	4.0 (12.1%)	15.0 (11.2%)	
2	17.0 (16.8%)	7.0 (21.2%)	24.0 (17.9%)	
6	0.0 (0.0%)	1.0 (3.0%)	1.0 (0.7%)	
**Macular status**				0.416^2^
Off	90.0 (89.1%)	31.0 (93.9%)	121.0 (90.3%)	
On	11.0 (10.9%)	2.0 (6.1%)	13.0 (9.7%)	
**Ocular comorbidities**				**0.018** ^**2**^
No	84.0 (83.2%)	21.0 (63.6%)	105.0 (78.4%)	
Yes	17.0 (16.8%)	12.0 (36.4%)	29.0 (21.6%)	
**Quadrants involved**				
**Superior-temporal**				0.319^2^
No	22.0 (21.8%)	10.0 (30.3%)	32.0 (23.9%)	
Yes	79.0 (78.2%)	23.0 (69.7%)	102.0 (76.1%)	
**Inferior-temporal**				0.131^2^
No	49.0 (48.5%)	21.0 (63.6%)	70.0 (52.2%)	
Yes	52.0 (51.5%)	12.0 (36.4%)	64.0 (47.8%)	
**Inferior-nasal**				0.248^2^
No	59.0 (58.4%)	23.0 (69.7%)	82.0 (61.2%)	
Yes	42.0 (41.6%)	10.0 (30.3%)	52.0 (38.8%)	
**Superior-nasal**				0.681^2^
No	54.0 (53.5%)	19.0 (57.6%)	73.0 (54.5%)	
Yes	47.0 (46.5%)	14.0 (42.4%)	61.0 (45.5%)	
**Total detachment**				0.089^2^
No	74.0 (73.3%)	19.0 (57.6%)	93.0 (69.4%)	
Yes	27.0 (26.7%)	14.0 (42.4%)	41.0 (30.6%)	

1. Wilcoxon rank test

2. Pearson’s Chi-squared test

Regarding the extent of RD (the involvement of different quadrants), we found no significant difference in recurrence rates between cases with superior-temporal (ST) (*p* = 0.319), inferior temporal (IT) (*p* = 0.131), superior-nasal (SN) (*p* = 0.681), or inferior-nasal (IN) (*p* = 0.248) quadrants involvement. In addition, patients with total detachment showed a higher recurrence rate (34.1% vs. 20.8%); however, it did not reach statistical significance (*p* = 0.089).

### Logistic regression analysis

We conducted multivariate analysis and logistic regression between rate of recurrence and the risk factors, which revealed that laterality [OR:3.81, 95%CI: 1.28–11.39], (*p* = 0.016), high myopia [OR:3.68, 95%CI: 1.23–11.00], (*p* = 0.02), and PVR [OR:8.42, 95%CI: 2.15–33.01], (*p* = 0.002) predicted the occurrence of redetachment in our cohort. However, the surgical technique and ocular comorbidities factors did not remain significant. (Table 4)

**Table 4:** Multivariate logistic regression of risk factors for recurrence of RD

Predictor	SE	Odds ratio	95% Confidence Interval		*P*-value
			Lower	Upper	
**Sex:**					
male–female	0.686	1.4139	0.3682	5.429	0.614
**Laterality**:					
right – left	0.558	3.8177	1.2788	11.397	**0.016**
**Surgical Technique**:					
PPV – SB	0.537	2.0433	0.7138	5.850	0.183
**Family History**:					
yes – no	0.960	0.5908	0.0900	3.880	0.584
**Smoking**:					
yes – no	0.675	0.6603	0.1760	2.477	0.538
**High myopia**:					
yes – no	0.558	3.6820	1.2323	11.002	**0.020**
**PVD**:					
yes – no	0.539	1.3307	0.4623	3.831	0.596
**PVR**:					
yes – no	0.697	8.4247	2.1497	33.016	**0.002**
**Lens Status**:					
aphakic – phakic	1.329	8.5759	0.6333	116.124	0.106
pseudophakic – phakic	0.620	1.9739	0.5854	6.656	0.273
**Number of Breaks**:					
2–1	0.687	0.7490	0.1950	2.876	0.674
3–1	0.898	0.8012	0.1380	4.653	0.805
**Macular Status**:					
on– off	1.083	0.3499	0.0418	2.925	0.332
**Ocular Comorbidities**:					
yes– no	0.589	1.6840	0.5314	5.337	0.376
**Total Detachment**:					
yes– no	0.647	0.7441	0.2092	2.646	0.648
**ST**:					
yes– no	0.605	0.4509	0.1377	1.476	0.188
**IN**:					
yes– no	0.753	1.5353	0.3511	6.714	0.569
**IT**:					
yes– no	0.687	0.3497	0.0910	1.343	0.126
**SN**:					
yes– no	0.606	0.7818	0.2384	2.564	0.685

## Discussion

More than 10% of RRD cases need additional interventions to repair the recurrent detachment [17]. Although there have been advancements in vitreoretinal surgery and tamponades, it is still crucial to identify risk factors for recurrence.

We found a recurrence rate of 24.6%, most of which were of early recurrence at less than 6 weeks after surgery. There is a continuous debate about the recurrence rate in SB compared to PPV; researchers thought SB might lead to higher rates of retinal redetachment because of the remaining pathologic vitreous body with its potential tractional forces. However, this study found that PPV (34.8%) had a borderline significantly higher recurrence rate than scleral buckling (19.3%) (*p* = 0.049). This might be due to the differences in training and preferences toward surgical technique choices, where our center offers junior ophthalmologists adequate training for SB. Jia et al. found a significantly higher incidence in the scleral buckling group (*p* = 0.031) [11]. Several studies found no difference in recurrence rates between vitrectomy and scleral buckling at six months [18, 19]. Similarly, a recent meta-analysis revealed nearly no difference in recurrence rate at 3–12 months after PPV or SB [20]. Further long-term investigation is needed to study the difference between PPV and SB regarding recurrence rates.

Regarding laterality, the right eye is more prone to retinal detachment. Our univariate analysis revealed a significantly higher right eye recurrence rate (*p* = 0.02). Also, multivariate analysis supported that the right eye has a 3.81-fold higher risk for recurrence than the left eye. Beheiri et al. found that the right eye had a 2.8 times more significant risk of retinal detachment than the left eye [21]. Conversely, Irigoyen et al. and Jia et al. found no significant difference between right and left eyes in the recurrence rate [11, 22]. 

Severe myopia could contribute to a higher risk of redetachment after surgical repair due to the elongation of the eyeball and thinning of the retina, making it more susceptible to tearing or detachment [23]. Our results found that eyes with severe myopia (≤ − 6.00 D) had higher recurrence (35.8%) than non-highly myopic eyes (17.3%) (*p* = 0.015). In addition, the multivariate analysis reported a significantly higher independent risk (OR of 3.68). Jia et al. had similar results with increasing recurrence rates in cases of longer axial length [11]. On the other hand, Irigoyen et al. showed no significant association between the recurrence of retinal detachment and myopia [22]. Despite conflicting results, the result of our study supports the more robust body of evidence that high myopia contributes to recurrence of RRD.

PVR is considered one of the leading causes of redetachment [24]. This study found a significant difference between recurrent and successful groups regarding PVR; 55% of recurrent cases had PVR, compared to only 19.2% of successful cases. Literature supports that PVR is a significant risk factor for recurrent retinal detachment; Ambiya et al. and Wickham et al. found a higher risk for redetachment with high grades of PVR [12, 25]. In addition, our multivariate analysis revealed a similar effect of PVR with an OR of 8.42. Interestingly, Lindsell et al. reported a much higher OR of 72.53 for PVR as a risk factor [26]. The inconsistent findings of different research indicate that the association between PVR and recurrent retinal detachment is complex and may be influenced by factors such as excessive myopia and a lack of severity grading. These confounding variables could explain the disparity in data on the risk of redetachment related to PVR. This sheds light on the importance of future randomized studies to account for this variability.

Regarding surgeons’ experience, our univariate analysis revealed that the recurrence rate of retinal detachment varied significantly among surgeons; the highest recurrence rate was associated with residents, while the lowest was with professors (*p* = 0.001). Numerous studies have shown similar results [22, 27, 28]. Nevertheless, multivariate analysis showed nonsignificant results with surgeons and comorbidities; this can be explained through different aspects like a significant number of cases with high myopia and surgeon experience that may correlate with case complexity, as residents handling simpler cases vs. professors managing high-risk PVR or inferior breaks. Also, information about tamponade choice was not reported.

Despite the common belief that certain demographic factors may influence the recurrence of retinal detachment, our analysis showed several demographic characteristics, including age, sex, family history, smoking, posterior vitreous detachment, number of retinal breaks, lens status, macular status, and retinal detachment quadrant involvement, did not show significant associations with recurrence.

Evidence supports that pseudophakic patients are more likely to get redetachment after surgical repair [11, 29]. This study found no significant association between lens status and redetachment. Most recurrent cases were phakic (57.6%), and 36.4% were pseudophakic, which can justify our findings. Although 57.6% of recurrent cases were smokers and 63.4% were successful cases, there was no significant difference between the two groups. A previous study found it associated with an increased retinal detachment surgery failure hazard [30]. 

Although the superior temporal quadrant was the most involved part, there was no difference between the recurrence and recurrence groups in all quadrants. A previous study showed a significantly higher recurrence linked to inferior detachment [22]. Though the number of retinal breaks is thought to be a potential risk for redetachment, most of our cases had one break in both groups, with no significant difference between them. Irigoyen et al. also found no significant association [22]. 

The association of macular status was debated in the literature; Irigoyen et al. and Cicineli et al. also found no significant association between redetachment and macula off [22, 31]. Posterior vitreous detachment increases the risk for initial retinal detachment. Still, there is no significant direct role in redetachment, which is consistent with our findings. However, there is an indirect thought that PVD can lead to PVR, leading to recurrent detachment [32]. 

Regarding gender, Irigoyen et al. stated that males have a 2 times higher risk for recurrence compared to women [22]. Furthermore, Callaway et al. and Jia et al. stated that women had a notable decreased risk of reoperation for redetachment of the retina. This could be explained by the fact that these results could be due to biological factors, such as abnormal adhesions in the vitreoretinal interface and longer axial lengths seen more in males [33]. Further studies will be required to analyze the impact of gender on retinal redetachment and further investigate the potential biological mechanisms behind these gender differences. We formulated a table comparing recurrence rates in our cohort in different population subgroups with literature represented by two systematic reviews and meta-analyses studies (Znaor et al. and He et al.) and a large sample primary study (Irigoyen et al.) [20, 22, 34] (Table 5).

**Table 5:** Recurrence rates in different subgroups compared to the literature

Variable, *n* (%)	Tawfik et al.	Znaor et al.	He et al.	Irigoyen et al.
Total Recurrence	33 (24.6%)	323 (24.4%)	548 (15.1%)	165 (28.9%)
Recurrence in PPV	16 (35.5%)	139 (21%)		161 (28.9%)
Recurrence in Scleral Buckle	17 (19.3%)	184 (28%)		10 (25.6%)
Recurrence in High Myopia	16 (35.8%)		91 (37.9%)	34 (34%)
Recurrence in pseudophakia	12 (33.3%)	80 (30.1%)	62 (25.6%)	76 (29.7%)
Recurrence in PVR	11 (55%)			27 (32.9%)
Recurrence in total RD	14 (34.1%)			17 (40.5%)
Recurrence in Males	25 (27.4%)			120 (33.1%)
Recurrence in Females	9 (20.9%)			45 (21.6%)
Recurrence in the Right eye	25 (33.7%)			99 (31.9%)
Recurrence in the Left eye	9 (15%)			260 (25.4%)

Nonetheless, it is known that retinal detachment is more common in people over 50 years old, and the included patients’ mean age was 48.9, with a mean of 47.3 in the recurrent group and 49.4 in the success group. No significant association was found between age and the incidence of recurrence. Similarly, Irigoyen et al. showed that there is no considerable difference between recurrence and non-recurrence groups [22]. On the contrary, a study revealed a notable association with age (OR = 0.972), validating that recurrence rates are often lower in younger people [11].

This study had some limitations that might affect the outcomes. Initially, the retrospective data collection approach, depending on current medical records, can result in insufficient information and possible bias. Also, there was a lack of information regarding the drugs selected for tamponade, intraoperative difficulties, or postoperative patient noncompliance during follow-up, which affects the recurrence of retinal detachment. This study lacked data about BCVA and another important measures due to incomplete patients record. Further studies should consider important preoperative measures in analysis. Also, further studies are warranted to stratify RRD types and identify differences. Additionally, a comparison of recurrence rates in patients undergoing supplementary SB following PPV with isolated PPV and SB was not applicable in this study. This should guide future research. The six-month follow-up period limits this study, as there is a potential for recurrence beyond this period, particularly in high-risk eyes. This study also failed to stratify PVR severity, restricting the analysis of the influence of those factors on recurrence. The variation in surgeons’ experience may limit the outcomes, as the procedures were conducted at a single center. It’s essential to have multicenter prospective studies to validate findings in other settings and assess the surgeon’s experience and RRD recurrence rate at different centers.

## Conclusion

In this study, the recurrence rate following surgical repair of RRD was 24.6%, with pars plan demonstrating a borderline higher recurrence rate compared to scleral buckling. Right eye laterality, high myopia, and PVR were identified as independent predictors of recurrence, while age, sex, and lens status showed no significant associations. Our findings support the current evidence that neither PPV nor SB offers advantages in preventing recurrence across all patient subgroups, highlighting the importance of individualized surgical planning. Further long-term randomized controlled trials and systematic reviews are needed to clarify the influence of surgical technique on recurrence, particularly across different types of RRD.

## Data Availability

The datasets analyzed during the current study are available from the corresponding author on reasonable request.

## Abbreviations

CI: Confidence Interval
IN: Inferior-Nasal (retinal quadrant)
IT: Inferior-Temporal (retinal quadrant)
OR: Odds Ratio
PPV: Pars Plana Vitrectomy
PVD: Posterior Vitreous Detachment
PVR: Proliferative Vitreoretinopathy
RRD: Rhegmatogenous Retinal Detachment
SB: Scleral Buckling
SD: Standard Deviation
SF6: Sulphur Hexafluoride (intraocular gas)
SN: Superior-Nasal (retinal quadrant)
SO: Silicone Oil (tamponade agent)
ST: Superior-Temporal (retinal quadrant)
